# Simple Determination of Gold Nanocrystal Dimensions by Analytical Ultracentrifugation via Surface Ligand-Solvent Density Matching

**DOI:** 10.3390/nano11061427

**Published:** 2021-05-28

**Authors:** Guillermo González-Rubio, Holger Hilbert, Rose Rosenberg, Bing Ni, Lisa Fuhrer, Helmut Cölfen

**Affiliations:** Physical Chemistry Department of Chemistry, University of Konstanz, Universitätsstraße 10, Box 714, 78457 Konstanz, Germany; holger.hilbert@uni-konstanz.de (H.H.); rose.rosenberg@uni-konstanz.de (R.R.); bing.ni@uni-konstanz.de (B.N.); lisa.fuhrer@uni-konstanz.de (L.F.)

**Keywords:** analytical ultracentrifugation, size distribution, surface ligand, gold nanocrystals, density matching

## Abstract

Analytical ultracentrifugation (AUC) is a powerful technique to observe colloidal nanocrystals (NCs) directly in solution and obtain critical information about their physical-chemical properties. Nevertheless, a more comprehensive implementation of AUC for the characterisation of such a class of crystalline colloids has been traditionally impaired by the requirement of having a priori knowledge of the complex, multilayered structure formed by NC in solution. This includes the nature (density and mass) of the surface ligands (SLs) that provide NC colloidal stability and the shell of solvent molecules formed on it. Herein, we propose a methodology to determine the NCs size by using SLs with a density equal to that of the solvent. Thereby, the buoyancy force of the SL shell is neutral, and the density of the NCs is sufficient a priori knowledge to calculate their related mass and size distributions. The simplicity and reliability of the method are evaluated with cetyltrimethylammonium bromide (CTAB) stabilized spherical gold NCs (AuNCs) of dimensions ranging from 1 to 17 nm. The proposed method has great potential to be transferred to any non-crystalline and crystalline colloids of different nature and composition, which have a density that is equal to the bulk and can be stabilized by SLs having a density that matches that of the solvent.

## 1. Introduction

Observation and measurement are primary processes of natural science, critical for the development, testing, and modification of hypotheses and the advancement of modern technologies. Characterisation techniques enabling such processes are essential to increase understanding and knowledge in medicine, chemistry, biology, or physics. One of the areas where this is very relevant is in the field of colloidal NC science, where advanced characterisation techniques are required to investigate the tremendous diversity of the submicrometer structures with distinct compositions (e.g., metals, metal oxides, sulfides, selenides, etc.), shapes (e.g., spheres, rods, stars, triangles, etc.), and properties (e.g., magnetic, electric, optical) that are nowadays available [1,2,3,4,5]. In this context, the in situ characterisation of colloidal NC systems is undoubtedly desired, as it is often different from that in the dry state. For instance, most biomedical applications of NCs depend on their surface chemistry, structure, and aggregation state. Indeed, the NC colloidal stability is typically achieved by steric and electrostatic repulsions arising from the adsorption of ligands on the surface. In solution, colloidal NCs are in the form of core-shell structures where the NC locates at the core and the shell is composed by SLs. An additional shell may form, especially in aqueous systems, due to the adsorption of solvent molecules that form a solvation shell (SS). As a result, the characterisation of the complex structures formed by NC in solution (NC@SL@SS) is critical for understanding NC behaviour in solution [6,7].

Analytical ultracentrifugation is a unique characterisation technique capable of determining the size and mass distributions of different colloids such as proteins, polymers, and latex nanoparticles directly in solution with ångström resolution [8,9,10,11,12,13,14]. Moreover, it can provide information about their aggregation state and the population of different assemblies [15,16]. Using high centrifugal forces, AUC can observe samples with different hydrodynamic properties with high statistical relevance: mass, size, density in solution (i.e., partial specific volume, which accounts for the total volume change of a sample that occurs upon addition of a mass unity to it), and frictional properties. As every molecule/particle is detected, the real mass-weighted distributions can be directly obtained from AUC sedimentation velocity experiments [17,18,19,20,21,22,23,24,25]. In the case of NC@SL@SS, significant advancements have been made in the use of AUC for their study. For instance, modern computation software such as UltraScan has enabled the density characterisation of highly heterogeneous NC@SL@SS colloids with unprecedented resolution (if the nanoparticles have a common spherical shape) [26]. In combination with the development of multiwavelength detectors, AUC can characterise broad size distribution NC@SL@SS colloids even if they display diverse optical properties [12,27,28]. For these reasons, AUC also holds great prospects for its use in the characterisation of colloidal NCs in solution.

However, it is worth noting that the impressive development of wet chemistry methods for NC synthesis in the last two decades has been generally not accompanied by the use of AUC for their study. Although AUC can provide accurate information about the NC@SL@SS, in many cases, knowledge about the NC dimensions is preferred, and such information can only be obtained if we possess information about:

1. *NC@SL density in solution*: In an AUC experiment, the sedimentation (s_NC@SL@SS_) and diffusion (D_NC@SL@SS_) of the entire NC@SL@SS colloids over time and under an applied centrifugal field are observed. Although s_NC@SL@SS_ and D_NC@SL@SS_ can be successfully retrieved from the AUC data, their translation into NC mass values still demands a priori knowledge of the density in the solution of the NC@SL (i.e., the partial specific volume of the NC@SL in solution). As this information is typically unknown unless other characterisation techniques are utilised to obtain it, the use of AUC for investigating colloidal NCs in detail becomes challenging.
2. *Density of the NC and SL, and mass of SL*. In general, the adsorption of SLs has a strong impact on the sedimentation behaviour of NCs, owing to their low density (e.g., ca. 1.1–1.5 g/cm^3^ for most organic ligands vs. 4–20 g/cm^3^ for many NCs such as noble metals, metal oxides, and sulfides). Because the NCs and the SL sediment together as an assembly, the lower density of the organic shell drags the sedimentation of the NCs (i.e., different buoyancy). The magnitude of such an effect depends on the density and relative mass of both the NC and the SL shell [11,12,29,30]. In general, the inorganic NC density in solution is the same as in the dry state, and the bulk density is used. In the case of SL, the partial specific volume of the free SL is typically used. However, a priori knowledge of SL mass is completely unknown in most cases, which becomes one of the major obstacles toward using AUC to characterise NCs.

As a result, only in some specific cases where the NC are spherical, the solvation degree is very low (i.e., SS can be neglected, typically in organic solvent and using low molecular weight SL), or the mass of SLs is minimal compared to that of the NC, the size of NCscan be determined via AUC. Herein, we aim at overcoming current limitations found in the AUC analysis of colloidal NCs. We propose a strategy to determine the dimension of colloidal NCs through SLs possessing equal density to that of the surrounding solvent. In such a case, SLs do not contribute to the sedimentation process (i.e., does not sediment or float), and only the NC is responsible for the sedimentation under an applied centrifugal field. However, because the SLs sediment together with the NC, they modify the frictional properties of the sedimenting NC. The effect of SL presence on the NC surface only impacts their NC diffusion properties, which can be determined from the AUC data. Using this ligand-solvent density-matching strategy, a priori knowledge of the NCs density (i.e., that of the bulk in many cases) is uniquely required to retrieve the NC mass from the AUC data analysis. This situation can be seen in similarity to Neutron Scattering using contrast matching conditions [31]. Here, solvent variations (usually achieved via H_2_O/D_2_O mixtures) are utilised to match the scattering contrast of one component in a multi-component species and thus characterise the properties of all other components except the one, which is matched out.

To test our hypothesis, we have investigated the sedimentation velocity behaviour of spherical AuNCs with different dimensions, ranging from 1 to 17 nm dispersed in water. As SLs, we have selected CTAB molecules, which form micelles with a density close to that of water at 20 °C, i.e., between 0.9711 and 0.99193 g/mL in the 1–15 mM range (i.e., differences ranging from ca. 2.6 to 0.6% with respect to water) [32]. Moreover, for the analysis of NCs with smaller dimensions and larger fractions of adsorbed surfactant and water (i.e., more sensitive to the deviation of the SL shell density from that of the water), the use of a co-surfactant, *n*-decanol, enables to precisely match the density of the CTAB micelles to that of water (i.e., 0.998220 g/mL).

## 2. Materials and Methods

### 2.1. Chemicals

All starting materials were used without further purification. Hexadecyltrimethylammonium bromide (CTAB 99+%,), hexadecyltrimethylammonium chloride (CTAC, 25% *w/w*, 756 mM), and sodium borohydride (NaBH_4_, 99%) were purchased from ACROS Organics (Antwerp, Belgium). Hydrogen tetrachloroaurate trihydrate (HAuCl_4_·3H_2_O, ≥99.9%), L-ascorbic acid (≥99%), deuterium oxide (99.9% D), toluene (ACS reagent, ≥99.5%), ethanol (anhydrous, 99.8%), (methanol (anhydrous, 99.8%), 1-dodecanethiol (>98%), and 1-decanol (*n*-decanol, 98%) were purchased from Merck (Darmstadt, Germany). MilliQ-grade water (resistivity 18.2 MΩ cm at 25 °C) was used in all experiments.

### 2.2. n-decanol/CTAB Solution

The growth solution was prepared by adding 9.111 g of CTAB (50 mM) and 870.5 mg (11 mM) of *n*-decanol to 500 mL of water and stirring the mixture at ~60 °C for 30–60 min, in a 500 mL Erlenmeyer flask.

### 2.3. Synthesis of 9 nm AuNCs

Au nanospheres were prepared by a seed-mediated approach as previously described in the literature [33], with some minor modifications: 50 μL of a 0.05 M HAuCl_4_ solution and 25 μL of a 0.1 M ascorbic acid solution were added to 5 mL of a 0.1 M CTAB solution under stirring at 27 °C. After 3 min, 200 μL of a freshly prepared 0.02 M NaBH_4_ (7.6 mg/20 mL) solution was injected under vigorous stirring. The mixture was kept undisturbed for 30 min at 27 °C. Then, an aliquot of 5 mL of the seed solution was added to 200 mL of 0.2 M CTAC solution, followed by 150 mL of a 0.1 M ascorbic acid solution. Subsequently, 200 mL of a 0.5 mM HAuCl_4_ solution was quickly injected under vigorous stirring. After 30 min, the resulting AuNCs were centrifuged for 1 h at 16,000 rpm, 24,320 rcf (using Hettich 1195-A 24 Place Angle Rotor in a Hettich MIKRO 220R, Tuttlingen, Germany) and redispersed in 25 mL of 25 mM CTAC solution. Then, the AuNCs were washed twice with a 2 mM CTAC solution before further use (by centrifugation in 2 mL Eppendorf tubes for 1 h at 16,000 rpm, 24,320 rcf). The final [Au^0^] concentration was adjusted to 4.16 mM (i.e., absorption at 400 nm of 1 when using a 0.1 cm optical path cuvette).

### 2.4. Synthesis of 13 and 17 nm AuNCs

Overgrowth of 9 nm AuNCs was found to be an optimal method to prepare 13 and 17 nm AuNCs. Such process was achieved by the addition of 2 mL (for 13 nm of final size) or 0.91 mL (for 17 nm of final dimensions) of the 9 nm AuNCs solution to 100 mL of a 25 mM CTAC solution, in the presence of 0.25 mM of HAuCl_4_ and 0.2 mM of ascorbic acid. The mixture was kept undisturbed for 1 h, washed twice by centrifugation (1 h at 12,000 rpm, 13,680 rcf), and redispersed in 2 mM CTAB solution. The final [Au^0^] concentration was adjusted to 4.16 mM (i.e., absorption at 400 nm of 1 when using a 0.1 cm optical path cuvette).

### 2.5. Synthesis of AuNCs with Dimensions Below 3 nm

Au nanospheres were prepared by a previously described method with minor modifications [34]: 25 mL of an *n*-decanol/CTAB solution was placed in a 50 mL glass beaker, and 250 µL of a 0.05 M HAuCl_4_ solution was added. After complete homogenization of the mixture by stirring for 5 min (300 rpm), 125 µL of a 0.1 M ascorbic acid solution was added. Then, the orange-yellow mixture slowly turned into a colourless solution. After 1–2 min, 250 µL of a freshly prepared 80 mM NaBH_4_ was injected under stirring (300 rpm using a PTFE plain magnetic stirring bar: 30 × 6 mm) at 30 °C, giving rise to a dark brownish-yellow solution.

### 2.6. Transmission Electron Microscopy

TEM images were obtained using a JEOL JEM-2200FS transmission electron microscope (Akishima, Tokyo) operating at an acceleration voltage of 200 kV. *TEM Grid Preparation of 1–3 nm AuNCs.* Due to their minimal dimension of the AuNCs, they are highly reactive, and the application of centrifugal forces conducts to their coalescence at the bottom of the centrifuge tube. Moreover, they are only stable in the presence of high surfactant concentrations. Thus, removing the excess of surfactant by centrifugation to enable their TEM imaging is not possible unless the surface of the seeds is passivated with strongly binding thiolated ligands. We used dodecanethiol to protect the AuNCs before removing the surfactant following a modified protocol previously described in the literature [35]. First, 1 mL of 2% dodecanethiol in ethanol (*v/v*) was added to 1 mL of seed solution and sonicated for 30 s. After 30 min incubation, the milky mixture was diluted with methanol until it became clear (ca. 3–4 mL) and centrifuged at 13,500 rpm (17,360 rcf) in 2 mL Eppendorf tubes for 10 min (using Hettich 1195-A 24 Place Angle Rotor in a Hettich MIKRO 220R centrifuge, Tuttlingen, Germany). Then, the supernatant was discarded, and the nanospheres were recovered from the Eppendorf walls using 300–400 µL of toluene. After adding 3.6 mL of methanol, the seeds were precipitated by centrifugation at 13,500 rpm (17,360 rcf) for 30 min (using Hettich 1195-A 24 Place Angle Rotor in a Hettich MIKRO 220R centrifuge) and redispersed in 200–300 µL of toluene. Finally, 20 µL of the seed solution was drop cast on a 2 nm carbon-coated 400 square mesh copper grid (QUANTIFOIL^,^ Jena, Germany) placed on a filter paper.

### 2.7. UV/Vis/NIR Spectra

All experiments were carried out using a Varian Cary 50 spectrophotometer (Santa Clara, CA, United States) at 298 K and quartz cuvettes with optical pathlengths of 0.1 cm.

### 2.8. Dynamic Light Scattering

DLS measurements were performed with a Zetasizer Nano from Malvern (Malvern, United Kingdom), and the data was evaluated with the Zetasizer Nano software v3.30 (Malvern, United Kingdom). Each sample was measured five times, and volume-weighted values are given.

### 2.9. Density and Viscosity of the CTAB and n-decanol/CTAB solutions

An Anton Paar density meter DMA 5000 M was used to determine the viscosity and density of the *n*-decanol/CTAB solutions at 20.00 °C used in all experiments. Briefly, 455 mg of CTAB and 43.5 mg of n-decanol were weighed in an empty 50 mL volumetric flask. Water was added to the flask to adjust the volume to the 10 mL mark. The mixture was heated to 40 °C and stirred for one hour until complete dissolution of the CTAB and *n*-decanol. The solution was left to cool down to room temperature for at least 10 h prior to any density measurements. The partial specific volume was calculated using the densitometric technique (1):

(1) $$\bar{v}=\frac{1}{\rho_{0}}\left(1-\left(\frac{\rho -\rho_{0}}{c}\right)\right),$$

where c is the concentration of the CTAB in g/mL, and ρ and ρ_0_ are the solution and solvent densities (g/mL), respectively.

### 2.10. Multiwavelength Analytical Ultracentrifugation

All AUC sedimentation velocity experiments were performed using a UV-Vis multiwavelength AUC (the general setup has been described in the literature: [28,36,37,38] equipped with an An-60 Ti Analytical 4-Place Titanium Rotor (Beckman Coulter) and 12 mm double sector cells (standard titanium centrepieces from Nanolytics, Potsdam, D) with quartz windows [27,28]. The nanosphere sedimentation behaviour was investigated at 20 °C (time for reaching temperature equilibrium was at least 60 min). Rotor speeds between 2000 and 4000 rpm were used to investigate the sedimentation behaviour of AuNCs with sizes between 9 and 17 nm and 54,000 rpm for those with dimensions below 3 nm. Radial absorbance data were collected at one scan/min and radial steps of 100 µm for the largest nanospheres and 50 µm for the smallest ones. The NC concentration was adjusted to ensure an optical density at 400 nm between 0.7 and 1. For the AUC experiments with 9, 13, and 17 nm spheres, 83.3 μL of the respective suspensions were diluted to 1 mL of a 1 mM CTAB solution and centrifuged for 1 h at 16,000, 13,000, and 10,000 rpm (24,320, 16,060, and 9500 rcf, respectively). Finally, they were redispersed in 1 mL of a 1 mM CTAB solution. Each sample was run at least three times, which serves to determine the reliability of the obtained data (given in the form of mean values of the retrieved hydrodynamic parameters and their standard deviations). In the experiments with 9 nm nanospheres, the final CTAB concentration was adjusted by redispersion of the precipitated nanospheres in 1 mL of water (for the 10 μM) and CTAB solutions with the desired final concentration. In the case of the AuNCs with dimensions below 3 nm, they were diluted to half of the initial concentration with water ([CTAB] = 25 mM and [*n*-decanol] = 5.5 mM). The sedimentation velocity data recorded at 400 nm of the 9–17 nm nanospheres were fitted using the Software SEDFIT (version 16.1c) [9]. The least-squares g*(s) model was used to fit the experimental data with a resolution of 100–300 grid points, time-independent and radial invariant noise, Tikhonov–Phillips regularization, and confidence level (F-ratio) of 0.683. A maximum entropy regularization and confidence level of 0.96 were used to fit the data to the c(s) and c(M) model (with a resolution of 100–300 grid points) and thereby determine the values M, f/f_0_, and s for the different AuNCs (i.e., using the density of gold 19.33 g/cm^3^). The 1–3 nm AuNCs MWL-AUC data was analysed using UltraScan [39]. The method two-dimensional spectrum analysis (2DSA, fitting time- and radially-invariant noise components, and meniscus position, followed by 50 Monte-Carlo iterations) and PCSA (Tikhonov regularization) with the straight line constrain [40] was applied using the density of gold 19.33 g/cm^3^ ($\bar{𝑣}$ = 0.0518 mL/g). Genetic algorithm optimization was finally applied to retrieve the final M, D, and f/f_c_ distributions and the corresponding standard deviations. The n-decanol/CTAB density and viscosity used for the analysis of the 1–3 nm AuNCs were 0.99822 g/mL and 0.01138 Ps.

## 3. Results

### 3.1. Analysis of Colloid Sedimentation Behaviour: A Priori Knowledge

The basic equation accounting for the sedimentation process of a sample in a centrifugal field is the Lamm equation, which describes the change of concentration as a function of time that occurs due to sedimentation and diffusion (2) [41,42]:

(2) $$\frac{\partial c}{\partial t}=D\left(\frac{\partial^{2}c}{\partial r^{2}}+\frac{1}{r}\frac{\partial c}{\partial r}\right)-\omega^{2}s\left(r\frac{\partial c}{\partial r}+2c\right)$$

with c = concentration, s = sedimentation coefficient, r = radial distance from rotation center, ω = angular velocity, and t = time. The sedimentation coefficient is related to the mass, density, and diffusion coefficient (and thus frictional properties) through the Svedberg Equation (3):

(3) $$s=\frac{M\cdot D\cdot \left(1-\frac{\rho }{\rho_{P}}\right)}{\mathrm{RT}}\;,$$

with M and ρ_P_ as the molecular weight and density of the sedimenting solute in solution, respectively, ρ = density of the solvent, and R = gas constant. The frictional properties are then retrieved from the diffusion coefficient, D, which is related to the frictional coefficient, f, through the Stokes–Einstein equation for diffusion in solution (4) [42,43]:

(4) $$D\;=\frac{k\cdot T}{f}=\frac{R\cdot T}{N_{A}\cdot f}\;,$$

where k is the Boltzmann constant, R is the gas constant, N_A_ is the Avogadro’s number, and T the absolute temperature.

The determination of s and D (i.e., and subsequently f) distributions requires a closed analytical solution for the Lamm Equation (2). Due to the infeasibility of analytically solving it, computational modelling can be used instead to fit approximate solutions to a set of experimental data (using computer software such as SEDFIT or UltraScan) [39,44]. The described modelling can be performed by providing initial guess values of specific hydrodynamic parameters that are provided, which are: the range of s and D. However, to obtain M or ρ_P_ from the retrieved s and D values, a priori knowledge of one of them is required, as can be deduced from the Svedberg Equation (3).

### 3.2. Forces during Sedimentation Experiments: The Density-Matching Approach

**Figure 1:**
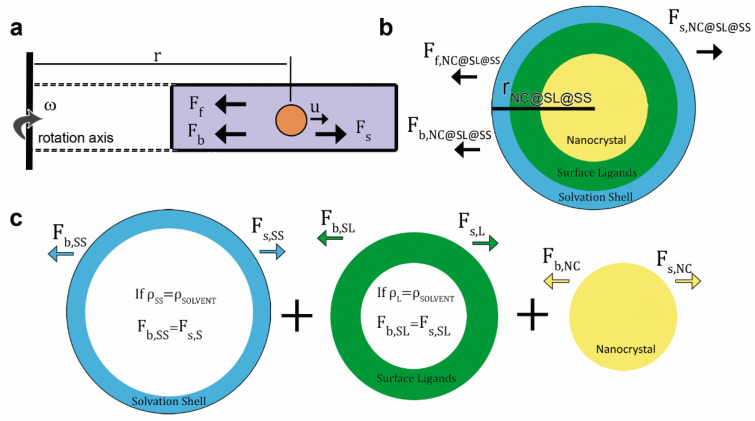
(**a**) Schematic of the three different forces acting on colloids during sedimentation under a centrifugal field. (**b**) Schematic of the model typically used to describe NCs colloidally stabilized by SLs and covered by a SS, and often applied to the AUC data analysis (r_NC@SL@SS_ = radius of the NC@SL@SS). (**c**) Schematic of the different centrifugal (F_s_) and buoyant (F_b_) force contributions arising from SSs (F_s,SS_ and F_b,SS_), SLs (F_s,SL_ and F_b,SL_), and NCs (F_s,NC_ and F_b,NC_). When the density of SLs (ρ_SL_) and SS (ρ_SS_) equals that of the solvent (ρ = ρ_SS_ = ρ_SL_), they do not float or sediment under an applied centrifugal field, as the centrifugal and buoyant forces reach equilibrium and cancel out each other: F_s,SL_ = F_b,SL_ and F_s,SS_ = F_b,SS_.

In an AUC experiment of NC@SL@SS, the interplay of three forces governs the sedimentation behaviour: (i) the applied centrifugal force (F_s,NC@SL@SS_), (ii) the buoyant force (F_b,NC@SL@SS_), and (iii) the frictional force (F_f,NC@SL@SS_ Figure 1a) [42].

F_s,NC@SL@SS_ is induced by the acceleration of the rotor (ω^2^r, ω = angular velocity, r = radius) and is proportional to the mass of NC@SL@SS (m_NC@SL@SS_, colloids with larger mass sediment faster). The buoyant force appears due to the displacement of the solvent by NC@SL@SS, being proportional to the solvent to solute density ratio (ρ/ρ_NC@SL@SS_). The frictional force also opposes the sedimentation due to the resistance of the solvent molecules to be displaced. It depends on the size, morphology, surface roughness, and solvation of the sample (reflected in the frictional and diffusion coefficients, f_NC@SL@SS_ and D_NC@SL@SS_) and the speed of sedimentation (u, i.e., terminal velocity) (5)–(7):

(5) $$F_{s,\mathrm{NC}@\mathrm{SL}@\mathrm{SS}}=\omega^{2}\cdot r\cdot m_{\mathrm{NC}@\mathrm{SL}@\mathrm{SS}}$$

(6) $$F_{b,\mathrm{NC}@\mathrm{SL}@\mathrm{SS}}=-\omega^{2}\cdot r\cdot m_{\mathrm{NC}@\mathrm{SL}@\mathrm{SS}}\cdot \frac{\rho }{\rho_{\mathrm{NC}@\mathrm{SL}@\mathrm{SS}}}$$

(7) $$F_{f,\mathrm{NC}@\mathrm{SL}@\mathrm{SS}}=-f_{\mathrm{NC}@\mathrm{SL}@\mathrm{SS}}\cdot u=-\frac{R\cdot T}{N_{A}\cdot D_{\mathrm{NC}@\mathrm{SL}@\mathrm{SS}}}\cdot u\;$$

The multilayered structure of NC@SL@SS, implies that the centrifugal and buoyancy force possess different contributions, arising from the distinct masses and densities of each layer (8) and (9):

(8) $$F_{s,\mathrm{NC}@\mathrm{SL}@\mathrm{SS}}=\omega^{2}\cdot r\cdot \left(m_{\mathrm{NC}}+m_{\mathrm{SL}}+m_{\mathrm{SS}}\right)$$

(9) $$F_{b,\mathrm{NC}@\mathrm{SL}@\mathrm{SS}}=-\omega^{2}\cdot r\cdot \rho \cdot \left(m_{\mathrm{NC}}\cdot \frac{1}{\rho_{\mathrm{NC}}}+m_{\mathrm{SL}}\cdot \frac{1}{\rho_{\mathrm{SL}}}+m_{S}\cdot \frac{1}{\rho_{\mathrm{SS}}}\right)$$

with m = mass and ρ = density, and the subscripts NC = nanocrystal, SL = surface ligand, and SS = solvation shell. Solvent density is given as ρ.

During the sedimentation experiment, a balance between the three forces is reached (10):

(10) $$F_{s,\mathrm{NC}@\mathrm{SL}@\mathrm{SS}}+F_{b,\mathrm{NC}@\mathrm{SL}@\mathrm{SS}}=-F_{f,\mathrm{NC}@\mathrm{SL}@\mathrm{SS}}$$

and by insertion of (4), (7), (8), and (9) in (10), we arrive at the Svedberg equation for NC@SL@SS (11, see SI for more details):

(11) $$s_{\mathrm{NC}@\mathrm{SL}@\mathrm{SS}}=\frac{D_{\mathrm{NC}@\mathrm{SL}@\mathrm{SS}}\cdot N_{A}\cdot \left[m_{\mathrm{NC}}\cdot \left(1-\frac{\rho }{\rho_{\mathrm{NC}}}\right)+m_{\mathrm{SL}}\cdot \left(1-\frac{\rho }{\rho_{\mathrm{SL}}}\right)+m_{\mathrm{SS}}\cdot \left(1-\frac{\rho }{\rho_{\mathrm{SS}}}\right)\right]}{R\cdot T}$$

In the vast majority of NC@SL@SS systems, ρ is equal to ρ_SS_, and therefore F_b,SS_ equals F_s,SS_, which implies that the buoyancy term for SS in Equation (11) is zero. As a result, SS only contributes to Ff,NC@SL@SS (Figure 1b,c) and the Svedberg equation can be expressed as a function of m_NC_, m_SL_, ρ_NC_, and ρ_SL_ (12):

(12) $$s_{\mathrm{NC}@\mathrm{SL}@\mathrm{SS}}=\frac{D_{\mathrm{NC}@\mathrm{SL}@\mathrm{SS}}\cdot N_{A}\cdot \left[m_{\mathrm{NC}}\cdot \left(1-\frac{\rho }{\rho_{\mathrm{NC}}}\right)+m_{\mathrm{SL}}\cdot \left(1-\frac{\rho }{\rho_{\mathrm{SL}}}\right)\right]}{R\cdot T}$$

From (12), it is evident that, once the values of s_NC@SL@SS_ and D_NC@SL@SS_ are determined by fitting the Lamm Equation (2) to AUC data, a priori knowledge of m_SL_, ρ_NC_, and ρ_SL_ is still required to determine m_NC_. For most inorganic NCs, it is possible to utilise the ρ_NC_ values of the bulk (their volume does not change from the dry to solvated state). In the case of ρ_SL_, it is typically assumed to be the same as the free ligand in solution (as determined from densitometry experiments) or pure ligand (in case of liquid ligands) [29,45]. However, a priori knowledge of m_SL_ is practically impossible, as it would already require possessing information about m_NC,_ which is the property of interest.

To overcome this issue, herein, we hypothesize that the AUC analysis of NC@SL@SS could be substantially simplified if ρ_SL_ is equal to ρ, as m_SL_ should only impact the frictional term of the sedimenting NC@SL@SS. The Svedberg equation would then be expressed as a function of the m_NC_ and ρ_NC_ (13)

(13) $${\;s}_{\mathrm{NC}@\mathrm{SL}@\mathrm{SS}}=\frac{D_{\mathrm{NC}@\mathrm{SL}@\mathrm{SS}}\cdot N_{A}\cdot m_{\mathrm{NC}}\cdot \left(1-\frac{\rho }{\rho_{\mathrm{NC}}}\right)}{R\cdot T}$$

and since M = m N_A_ (14):

(14) $$s_{\mathrm{NC}@\mathrm{SL}@\mathrm{SS}}=\frac{D_{\mathrm{NC}@\mathrm{SL}@\mathrm{SS}}\cdot M_{\mathrm{NC}}\cdot \left(1-\frac{\rho }{\rho_{\mathrm{NC}}}\right)}{R\cdot T}$$

or (15):

(15) $$M_{\mathrm{NC}}\cdot \left(1-{\bar{v}}_{\mathrm{NC}}\;\rho \right)=\frac{s_{\mathrm{NC}@\mathrm{SL}@\mathrm{SS}}\cdot R\;T}{D_{\mathrm{NC}@\mathrm{SL}@\mathrm{SS}}}$$

Through this ligand-solvent density-matching strategy, only a priori knowledge of ρ_NC_ (or partial specific volume ${\bar{v}}_{\mathrm{NC}}=1/\rho$_NC_) would be required to interpret s_NC@SL@SS_ and D_NC@SL@SS_, and retrieve M_NC_ (molar mass of NC), which is often the property of interest (e.g., in the field of NC synthesis).

### 3.3. AUC Characterisation of CTAB Stabilized AuNC

We employed spherical AuNCs stabilized with CTAB (AuNC@CTAB@W) micelles in water as model systems to demonstrate the proposed ligand-solvent density-matching approach due to their ease of synthesis, chemical stability, and suitable optical properties (i.e., UV-Vis optical detectors can be used to monitor their sedimentation). We selected the cationic surfactant CTAB, which has been widely employed in the synthesis of AuNCs and possesses a density that matches closely that of water, i.e., between 0.97110 and 0.99193 g/mL in the 1–15 mM range vs. 0.99822 g/mL for water at 20 °C, respectively. Thereby, we investigated the effect of different CTAB concentrations on the diffusion and frictional properties of the AuNCs, and their influence on the retrieved size distributions.

The frictional properties of sedimenting solutes are typically expressed in terms of the frictional ratio, f/f_0_. It is defined as the dimensionless ratio between the experimental frictional coefficient of the solute f and a non-solvated perfectly spherical (via the Stokes equation f_0_ = 6 π η r_0_) anhydrous particle of equivalent mass and partial specific volume of the solute f_0_ [46]. Squire and Himmel stated that f/f_0_ can be considered as the product of two terms: one term due to hydration and the other term due to shape [46].

However, because our solute of consideration is the NC, we substitute the friction f_0_ of the traditional anhydrous equivalent sphere by that of the spherical anhydrous NC core without any stabiliser f_c_ (reflected by the core radius r_c_) and obtain f/f_c_ instead of the traditional f/f_0_ (16):

(16) $$\frac{f}{f_{c}}=\frac{f_{\mathrm{NC}@\mathrm{SL}@\mathrm{SL}}}{f_{c}}$$

Since r_NC_ < r_NC@SL_, f/f_c_ > f/f_0_. From Equation (4) and the Stokes Equation (17):

(17) f=3π·η·d

The frictional ratio of NC@SL@SS, (f/f_c_)_NC@SL@SS_, with the NC as reference can be expressed as (18):

(18) $${\left(\frac{f}{f_{c}}\right)}_{\mathrm{NC}@\mathrm{SL}@\mathrm{SS}}=\frac{f_{\mathrm{NC}@\mathrm{SL}@\mathrm{SS}}}{f_{c}}=\frac{R\cdot T\cdot N_{A}^{-1}\cdot D_{\mathrm{NC}@\mathrm{SL}@\mathrm{SS}}^{-1}}{3\pi \cdot \eta \cdot d_{c}}$$

where d_c_ is the diameter of the NC, and D_AuNC@CTAB@W_ is the diffusion coefficient of AuNC@CTAB@W. The frictional ratio of AuNC@CTAB@W, (f/f_c_)_AuNC@CTAB@W_, would be expressed as (19):

(19) $${\left(\frac{f}{f_{c}}\right)}_{\mathrm{AuNC}@\mathrm{CTAB}@W}=\frac{f_{\mathrm{AuNC}@\mathrm{CTAB}@W}}{f_{\mathrm{SphAuNC}}}=\frac{R\cdot T\cdot N_{A}^{-1}\cdot D_{\mathrm{AuNC}@\mathrm{CTAB}@W}^{-1}}{3\pi \cdot \eta \cdot d_{\mathrm{SphAuNC}}}$$

where the index SphAuNC refers to the anhydrous spherical gold core in our particular case. Therefore, as CTAB does not contribute to the buoyant force, any change in the CTAB adsorbed on the AuNC surface should solely be reflected in (f/f_c_)_AuNC@CTAB@W_, D_AuNC@CTAB@W_, and s_AuNC@CTAB@W_ while the retrieved mass of the AuNC should be the same in all cases. It is worth noting that f/fc can be seen as a special case of f/f_0_ where f_0_ only considers the NC core, instead of the NC and the SLs, as it is typically done.

**Figure 2:**
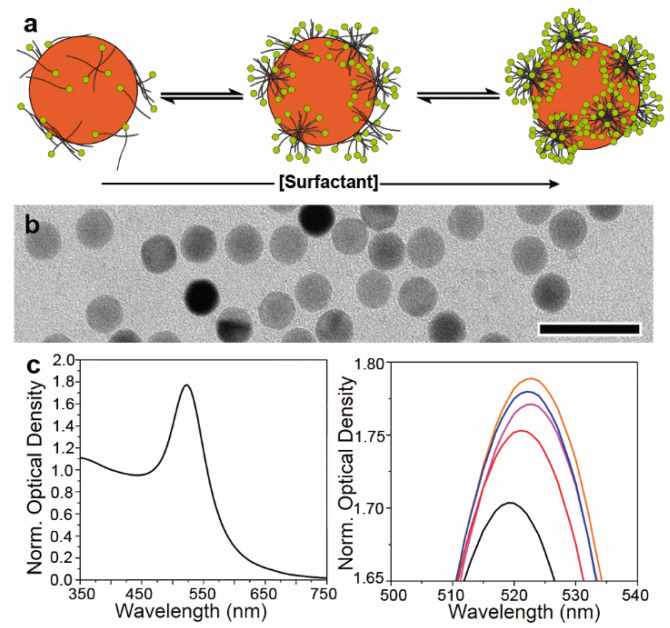
Tuning CTAB adsorption on AuNC surface. (**a**) Schematic view of the changes in the amount of CTAB adsorbed on the AuNC surface as a function of its concentration in solution. At concentrations below the CMC (ca. 1 mM for CTAB), molecules or small surfactant aggregates are expected to be adsorbed, while above the CMC, micellar structures are expected to be found on the metal surface. (**b**) TEM image of 9 nm AuNCs used to investigate the effect of surfactant adsorption on its sedimentation behaviour. Scale bar: 25 nm (**c**) Normalized UV-Vis spectra of 9 nm AuNCs (left) and the impact of increasing concentration of CTAB (right) on the position and intensity of the plasmonic band: 10 µM (black), 125 µM (red), 500 µM (magenta), 1000 µM (blue), and 3000 µM (orange). Normalization is performed at 400 nm, where the AuNC absorption is less sensitive to the plasmonic property changes [49].

To evaluate this hypothesis with AUC experiments, we first investigated the surfactant adsorption by monitoring the optical properties of 9 nm AuNCs in the presence of different CTAB concentrations (Figure 2). 

Firstly, the adsorption of surfactants on the surface of the NCs represents an equilibrium, and therefore varying their concentration in solution should enable fine control over the amount of the adsorbed CTAB. Thus, below the critical micelle concentration (CMC, i.e., 1 mM for CTAB), only CTAB molecules should adsorb, while at concentrations equal and above it, micellar aggregates should form, leading to a larger amount of adsorbed surfactant (Figure 2a) [47,48]. 

Secondly, the optical properties of AuNCs arise from the light excitation of conduction electrons and subsequent formation of collective oscillations localized at the interface between the metal and a dielectric medium (i.e., CTAB and water in our case). As a result, small changes in the dielectric constant of the surroundings lead to alteration of the absorption and scattering of light by AuNCs [50]. In our case, when the concentration of CTAB was increased from 10 to 125 and 500 µM, a shift of the plasmon band from 519 nm to 521 and 523 nm was noticed (Figure 2c). Such a redshift can be explained by the adsorption of more significant amounts of CTAB molecules, a material with a higher refractive index than that of water (1.44 vs. 1.33). Such an effect favours the displacement of the plasmon resonances towards longer wavelengths as well as the observed increase in their intensity [51]. Further improvement of the absorption without plasmon position modification was observed at concentrations between 1000 and 3000 µM of CTAB, indicating that the CTAB micelles almost saturated the NC surface.

**Figure 3:**
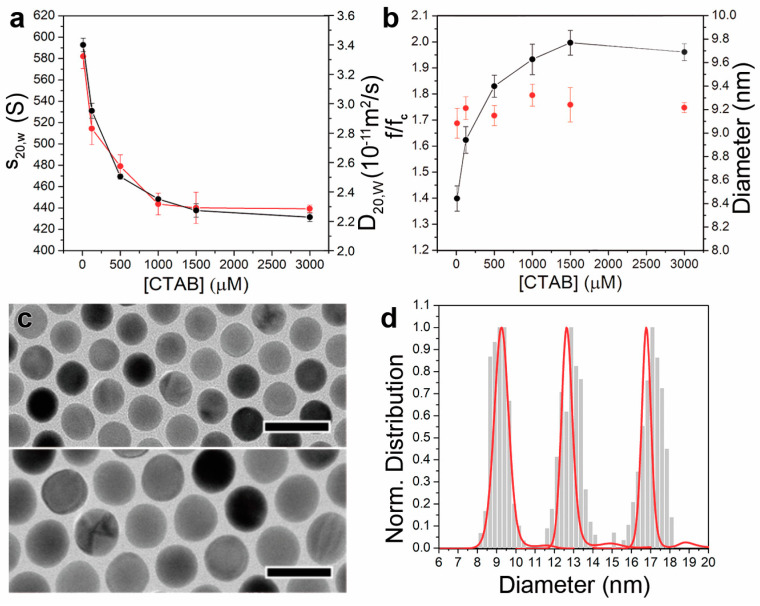
AUC determination of colloidal AuNC size distributions. (**a**) Variation of the s_20,w,AuNC@CTAB@W_ (black) and D_20,W,AuNC@CTAB@W_ (red, diffusion coefficient at standard conditions) of 9 nm AuNC as a function of the CTAB concentration. (**b**) Plot of the calculated (f/f_c_)_AuNC@CTAB@W_ (black) and dimension (red) of the 9 nm AuNCs at the different CTAB concentrations. (**c**). TEM images of AuNCs of 13 (top) and 17 nm (bottom) used in the AUC experiments. Scale bars: 25 nm. (**d**) Normalized size distributions of the 9, 13, and 17 nm AuNCs obtained from the analysis of TEM images (bars) and AUC data (red).

The changes in the amount of adsorbed CTAB were revealed even more clearly during sedimentation velocity AUC experiments, where a substantial shift of the AuNC@CTAB@W colloids sedimentation behaviour was observed (Figure 3a,b). The analysis of the AUC data was carried out using the continuous c(s) and c (M) distribution models implemented in Sedfit, and thereby we obtained the relevant data about s_20,w,AuNC@CTAB@W_ and M_AuNC@CTAB@W_ distributions, and weight-average frictional ratio and D_AuNC@CTAB@W_. The density of gold 19.33 g/cm^3^ was used to perform the analysis. 

A sharp decrease of the sedimentation coefficient (given at standard conditions, s_20,w,AuNC@CTAB@W_) from 593 to 526 and 469 S was induced when increasing the surfactant concentration from 10 to 125 and 500 µM, respectively (Figure 3a). Between 500 µM and 1500 µM of CTAB, the sedimentation velocity change was significantly reduced (i.e., from 469 to 430 S), whereas it remained almost constant when even higher CTAB concentrations were used (426 S).

The change in the sedimentation velocity could be thus attributed to an increase of (f/f_c_)_AuNC@CTAB@W_. Such friction would arise from the adsorption of hydrated CTAB micelles, which act as a parachute (Figure 1b). Consequently, D_AuNC@CTAB@W_ and (f/f_c_)_AuNC@CTAB@W_ were found to decrease and increase, respectively, together with the presence of CTAB in the solution. Such observation was transduced into an increment of the hydrodynamic diameter (d_AuNC@CTAB@W_) as obtained from the Stokes Equation (17) (if we can assume that the entire AuNC@SL@W has a spherical shape in solution), from 12.6 to 18.3 nm when the CTAB concentration was raised from 10 µM to 1000–3000 µM (Table 1 and Table 2).

It is worth noting that due to the spherical morphology of the investigated AuNCs, it is possible to transform the AuNC mass values obtained from the AUC data analysis (Equation (13)) into dimensions directly (Equations (20) and (21), Table 2):

(20) $$d_{\mathrm{SphAuNC}}=2\cdot r_{\mathrm{SphAuNC}}=\cdot 2\left(\sqrt[{3}]{\frac{3\cdot V_{\mathrm{SphAuNC}}}{4\cdot \pi }}\right)$$

(21) $$V_{\mathrm{SphAuNC}}=\frac{m_{\mathrm{SphAuNC}}}{\rho_{\mathrm{SphAuNC}}}$$

where d_SphAuNC_, m_SphAuNC_, and ρ_SphAuNC_ are the diameter, mass, and density of the spherical AuNCs.

The AuNC dimensions calculated from the AUC experiments performed in different CTAB concentrations were found to differ by less than 2% from those obtained via the TEM images analysis (Figure 3c,d and Table 2). Thus, the obtained results strongly support the suitability of the hypothesis proposed in this work. The use of the CTAB, with ρ_CTAB_ very close to that of water (differences ranging from 2.6% to 0.6% in the 1–15 mM range), enables direct retrieving of the AuNC dimensions (i.e., using ρ_Au_ as a priori knowledge for AUC data analysis). From the analysis of the AuNC@CTAB@W sedimentation data, it is thereby possible to determine M_AuNC_ (i.e., the only component that possesses buoyant mass). Then, through D_AuNC@CTAB@W_, we can determine d_AuNC@CTAB@W_, while from the M_AuNC_, we obtain d_AuNC_ (Table 1 and Table 2). The hydrodynamic diameter calculated by AUC clearly indicates that more CTAB adsorbs on the NC surface when its concentration increases. A similar trend was also observed via characterisation of d_AuNC@CTAB@W_ using dynamic light scattering, although the resolution is an order of magnitude lower than in AUC.

Finally, we calculated the thickness of the swollen CTAB shell t_CTAB@W_ from the difference between d_AuNC@CTAB@W_ (from Equations (4) and (17)) and d_AuNC_ from Equation (20) (Table 2) (Equation (22)):

(22) $$t_{\mathrm{CTAB}@W}=\;\frac{d_{\mathrm{AuNC}@\mathrm{CTAB}@W}-d_{\mathrm{AuNC}}}{2}$$

We found a consistent increase of the t_CTAB@W_ with the rise of the CTAB concentration, which can explain the decrease of the observed s_20,w,AuNC@CTAB@W_ and D_20,w,AuNC@CTAB@W_, and the increase of (f/f_c_)_AuNC@CTAB@W_ (i.e., f_AuNC@CTAB@W_ increases but f_SphAuNC_ remains constant, Table 1).

The proposed methodology was further evaluated to investigate its suitability for the characterisation of AuNC colloids with larger dimensions. Thus, AuNCs of 13 nm and 17 nm were synthesized, and their size distributions were determined via AUC following the described approach (in this case, in the presence of 1 mM of CTAB). As observed in the 9 nm AuNC case, the deviation of d_AuNC_ values from those obtained by TEM analysis was found to be below 2% (Figure 2b and Figure 3d). Not surprisingly, a decrease of (f/f_c_)_AuNC@CTAB@W_ (i.e., 1.68 ± 0.02 and 1.47 ± 0.05 for the 13 and 17 nm AuNCs, respectively) was observed as d_AuNC_ increased (i.e., larger NCs have a lower surface-to-volume ratio and therefore lower proportion of adsorbed CTAB to the Au mass). Moreover, the variation of the frictional properties followed the same trend noticed for the 9 nm AuNCs. When the presence of CTAB in solution increased from 10 µM to 3 mM, (f/f_c_)_AuNC@CTAB@W_ of the 13 nm AuNCs was found to increase from 1.34 to 1.74, respectively. In the case of the 17 nm AuNCs, such variation occurred from 1.07 to 1.48.

Nonetheless, the investigated Au colloids can be seen as ideal systems due to the low size dispersity and spherical morphology. An excellent example of a more complex system can be found in the Au seeds that are typically used to synthesize anisotropic AuNCs [4,52]. They are small unstable AuNCs of 1–2 nm, difficult to characterise by TEM, and covered with large amounts of CTAB (i.e., between 50 and 100 mM). For these reasons, AUC holds unique potential for investigating such types of unstable NC systems, providing the opportunity to observe their dimensions close to synthesis conditions.

**Figure 4:**
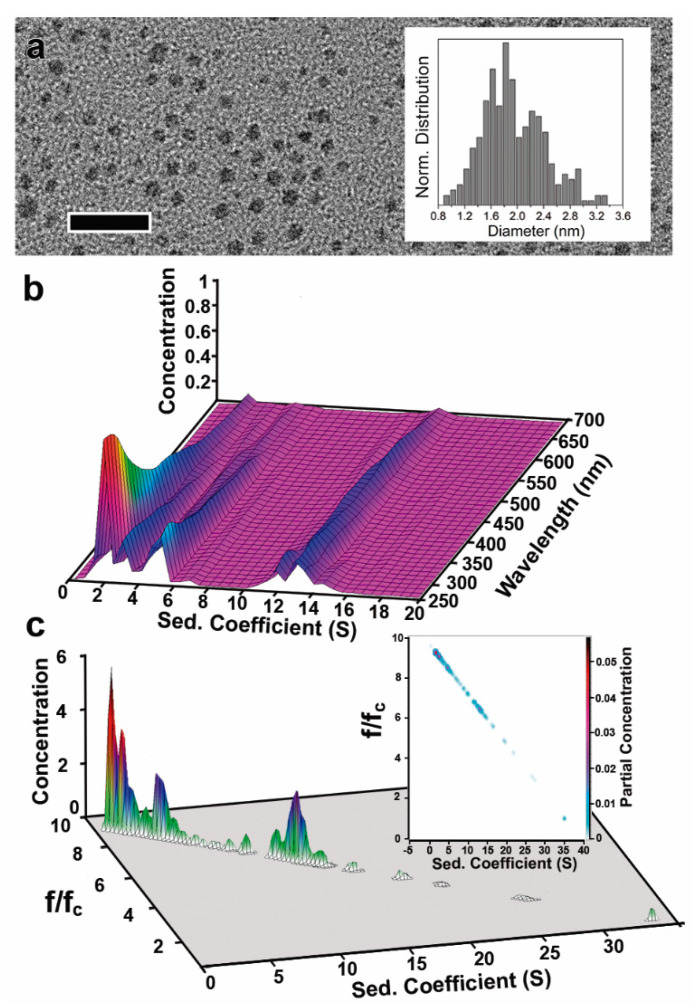
AUC size distribution determination of 1–3 nm AuNCs in the presence of high surfactant concentrations. (**a**) TEM image of AuNCs with dimensions of 1.7 ± 0.4 nm (inset). Scale bar: 12 nm. (**b**) 3D plot of the MWL-AUC analysis of the AuNCs where the s_AuNC@CTAB@W_ is determined for each wavelength of the 250–700 nm range. This reveals the species found dependent on the wavelength that they are absorbing. (**c**) 3D and 2D (inset) plots of sedimentation coefficient and (f/f_c_)_AuNC@CTAB@W_ (lower) distributions obtained from sedimentation velocity AUC measurements at 450 nm (using the ρ_Au_ to perform the analysis).”

Herein, we prepare a suspension of small AuNCs following a recently reported approach to synthesize Au nanorods (see Materials and Methods) [34]. The analysis by TEM only revealed a single broad size distribution, ranging from 0.8 nm to 3.3 nm (i.e., 1.7 ± 0.4 nm, Figure 4a), and pseudospherical morphologies in some cases, most probably due to the coalescence of the unstable small AuNCs during the TEM grid preparation and/or under the electron beam. These effects can be explained by the low stability of such small AuNCs. The need to deposit the AuNCs on special supports and remove the solvent to enable their characterisation by TEM can easily induce their coalescence.

To overcome this issue, strongly binding ligands containing thiol moieties (e.g., dodecanethiol) were used to stabilize the small AuNCs before purification and removal of the CTAB. Unfortunately, ripening effects and subsequent changes in the size distribution can be caused by the presence of thiols. Moreover, the TEM electron beam can decompose the protecting thiolated ligand shell, inducing the coalescence of the small NCs into non-spherical NCs of larger dimensions. Thus, TEM characterisation of such small AuNCs is challenging, and only a part of the sample can be observed using this technique, which compromises the statistical relevance of the results.

For the AUC experiments, the CTAB concentration during the sedimentation experiments was 25 mM, and *n*-decanol (5.5 mM) was added to match more precisely the density of the CTAB micelles to that of water (i.e., 0.998220 ± 0.000003 g/mL), as the contribution of the surfactant shell is expected to be much larger in such small AuNCs [32]. The viscosity, however, increases up to 0.01136 P in the presence of *n*-decanol. Moreover, *n*-decanol increases the stability of the small NC (i.e., it is a co-surfactant that increases the rigidity of CTAB micelles and delays the coalescence of the NC into larger ones, a common issue with this kind of unstable NC) [53].

However, the analysis of this type of small AuNC using AUC is also challenging. Small AuNCs (i.e., Au clusters) may possess different extinction coefficients depending on their size, and therefore, the analysis of their sedimentation behaviour may not be possible by measuring it at a single wavelength as in the case of 9 nm AuNCs [54]. For this reason, we characterised the AuNCs sedimentation in the UV-Vis range using the multiwavelength-AUC (MWL-AUC) and the data was analysed using the advanced UltraScan software package (from 250 to 700 nm; Figure 4b,c) [39]. 

First, the two-dimensional spectrum analysis was utilised to fit the noise and then the parametrically constrain optimisation method, PCSA, with the straight line constrain (which provides the lowest RMSD) was applied to determine the frictional ratio variation as a function of s. Genetic algorithm optimisation was finally applied to retrieve the final M_AuNC@CTAB@W_, D_AuNC@CTAB@W,_ s_AuNC@CTAB@W_, and frictional ratio distributions. The density of gold 19.33 g/cm^3^ was again used to perform the analysis, meaning that the determined frictional ratio is f/f_c_.

A broad AuNC size dispersity was quickly captured by MWL-AUC measurements (as reflected by the presence of five main bands in the s distribution plot, Table 3 and Figure 4b,c). All different species were found to absorb light in the 250–700 nm range. In this case, the precise determination of the AuNC dimension was performed at 450 nm, revealing the presence of AuNCs of 1.2, 1.5, 1.6, 2, and 2.86 nm with different concentrations (Figure 4c and Table 3). At different wavelengths (e.g., 360, 400, and 500 nm, Figure S1), similar s_AuNC@CTAB@W_ vs. (f/f_c_)_AuNC@CTAB@W_ distributions were also observed (see Supporting Information). These results suggest that in the 1–3 nm range, the optical properties of AuNCs are not significantly different.

While (f/f_c_)_AuNC@CTAB@W_ of AuNC with dimensions between 9 and 17 nm is below 2, decreasing the AuNC size down to the 1–3 nm range in the presence of high surfactant concentrations leads to a substantial increment of the (f/f_c_)_AuNC@CTAB@W_ up to 9.9. These results strongly suggest that for the small AuNCs, the influence of adsorbed CTAB on the sedimentation behaviour increases compared to the 9 nm ones, most probably due to the adsorption of a large amount of CTAB@W per Au NC unit mass. In fact, the mass of CTAB@W adsorbed (as calculated from the known thickness of the shell and its density, equal to that of water) on the 9 nm core varies from 0.009 to 0.035 g per gram of Au when CTAB concentration increases from 10 to 3000 µM. However, in the case of the 1–3 nm AuNCs, the mass of CTAB@W decreases from 27.7 to 9.34 g per gram of Au when the size increases from 1.2 to 2.9 nm.

These results strongly support the suitability of the ligand-solvent density-matching strategy for the characterisation of colloidal AuNCs, regardless of their dimensions. The use of co-surfactants such as *n*-decanol also emerges as an extremely interesting approach to potentially tune the density of surfactant micelles that stabilize the AuNCs and precisely match that of water, especially in the case of very small NCs with a large amount of adsorbed surfactants with respect to their mass. Moreover, in AuNCs with broad size dispersities, MWL-AUC enables proper determination of the wavelength required to characterise their sedimentation behaviour.

## 4. Conclusions

Overall, we have proposed an approach to overcome current issues limiting the use of AUC to characterise colloidal NCs stabilized by SLs and possessing thick SS shells. While existing methods typically require a priori knowledge of the density of the entire NC@SL@SS (and consequently that of their constituents), we circumvent this limitation by a ligand-solvent density-matching strategy employing SL, which has a density that matches that of the solvent. This approach implies that only the values of the ρ_NC_ are required to treat the AUC data, and therefore the dimensions of the NCs are directly retrieved. The presence of SLs is thus reflected solely on the frictional properties of the colloidal NC@SL. Using ρ_NC_ in the evaluation of the frictional ratio, f/f_0_ changes the reference system of the anhydrous equivalent sphere with the same mass and partial specific volume as the solute with friction f_0_ to the friction of the spherical anhydrous NC without the stabilizer shell f_c_ [46]. In case of a core-shell nanoparticle, the reference system of the pure spherical core is more practical since the frictional ratio f/f_c_ then directly expresses the influence of shape as well as the stabilizer and hydration shell onto the nanoparticle friction.

To demonstrate our new evaluation methodology, we have first investigated 9-nm AuNCs covered with different amounts of CTAB, a surfactant having a density matching that of water. Regardless of the amount of CTAB adsorbed on the AuNC, the analysis of the sedimentation velocity AUC data provided NC sizes in close agreement with those obtained from TEM analysis when the density of Au was used as a priori knowledge. As expected, significant variations of the sedimentation, diffusion, and frictional coefficients were observed as a function of the mass of adsorbed CTAB. Similar results were obtained for AuNCs of 13 and 17 nm. Finally, the generality and performance of the method were evaluated by testing the broad size dispersity of AuNCs with dimensions below 3 nm and dispersed in concentrated CTAB solutions. We were able to accurately determine the size distribution of the small AuNCs with angstrom resolution.

Thus, the reported strategy emerges as a robust method to characterise NC colloids stabilized by SLs in a radically simple and accurate manner from sedimentation velocity AUC data. This approach is especially suitable in the case of NC dimensions below 3 nm that are difficult to characterise by other means. Moreover, the described strategy might be capable of dealing with any hard NC colloids if the ρ_SL_ is equal to that of the solvent and the ρ_NC_ is known. Overall, it implies a great potential for the application of AUC for the characterisation of an array of inorganic colloids, including anisotropic nanocrystals, a type of nanomaterial hard to characterise by AUC.

The range of our density-matching method might even extend to the analysis of polymer complexes of interacting polymers as long as the density of one of the polymers can be matched by the solvent since the Svedberg equation for multi-component samples in Equation (9) can be applied to any kind of sample. This limits the range of suitable polymers if water as well as its mixtures with heavy water are applied as solvents. The solvent density can be increased by the dissolution of a small molecule or heavy salt, and that way, even the density of proteins could be matched. However, since these dissolved molecules or ions will form density gradients at higher speeds, this would complicate the evaluation and limit the analysis to large samples, which already create sediment at low speeds.

How far ligand density variations from that of the solvent are tolerated by our methodology remains to be explored in a future study, as well as the suitability of NC shapes deviating from that of a sphere.

## Figures and Tables

**Table 1:** Data at 20 °C obtained from the analysis of sedimentation velocity behaviour of 9 nm AuNCs using the density of Au as a priori knowledge: s_20,w,AuNC@CTAB@W_, D_20,w,AuNC@CTAB@W_, and (f/f_c_)_AuNC@CTAB@W_, and molar mass of AuNC (M_AuNC_).

[CTAB] µM	s_20,w,AuNC@CTAB@W_ (S, 10^−13^ s)	D_20,w,AuNC@CTAB@W_ (10^−11^ m^2^/s)	(f/f_c_)_AuNC@CTAB@W_	M_AuNC_ (MDa)
10	590 ± 7	3.32 ± 0.08	1.40 ± 0.05	4.5 ± 0.2
125	526 ± 4	2.8 ± 0.1	1.62 ± 0.05	4.8 ± 0.2
500	469 ± 4	2.58 ± 0.07	1.83 ± 0.05	4.8 ± 0.2
1000	447 ± 2	2.32 ± 0.07	1.93 ± 0.06	4.8 ± 0.2
1500	430 ± 1	2.3 ± 0.1	2.00 ± 0.05	4.8 ± 0.2
3000	426 ± 6	2.29 ± 0.02	1.96 ± 0.03	4.9 ± 0.1

**Table 2:** Data obtained from the analysis of sedimentation velocity behaviour of 9 nm AuNCs using the density of Au (ρ_Au_) as a priori knowledge: hydrodynamic radius (from AUC and DLS), and AuNC size, and the thickness of hydrated CTAB and water shells (t_CTAB@W_).

[CTAB] µM	dAuNC@CTAB@W by AUC (nm)	dAuNC@CTAB@W by DLS (nm)	dAuNC by AUC (nm)	dAuNC by TEM (nm)	tCTAB@W by AUC (nm)
10	12.6 ± 0.3	13 ± 4	9.1 ± 0.1	9.1 ± 0.4	1.9 ± 0.2
125	14.8 ± 0.6	14 ± 5	9.21 ± 0.09		2.4 ± 0.3
500	16.3 ± 0.5	15 ± 5	9.16 ± 0.09		2.9 ± 0.2
1000	18.1 ± 0.6	16 ± 4	9.32 ± 0.09		3.3 ± 0.2
1500	18.3 ± 0.8	16 ± 5	9.2 ± 0.1		3.4 ± 0.2
3000	18.3 ± 0.2	16 ± 3	9.22 ± 0.05		3.4 ± 0.2

**Table 3:** AuNC data obtained from the analysis of their sedimentation velocity behaviour at 450 nm.

s_20,w,AuNC@CTAB@W_ (S, 10^−13^ s)	D_20,w,AuNC@CTAB@W_ (10^−11^ m^2^/s)	d_AuNC@CTAB@W_ (nm)	(f/f_c_)_AuNC@CTAB@W_	M_AuNC_ (kDa)	d_AuNC_ (nm)	Rel. Conc. %
1.8 ± 0.1	3.8 ± 0.2	9.9 ±0.5	9.23 ± 0.05	11 ± 2	1.2 ± 0.2	25.6
2.6 ± 0.2	3.3 ± 0.1	11.5 ± 0.4	9.06 ± 0.05	19 ± 2	1.5 ± 0.2	18.1
3.6 ± 0.3	2.9 ± 0.1	12.8 ± 0.4	8.9 ± 0.1	28 ± 4	1.6 ± 0.2	11.0
5.2 ± 0.3	2.5 ± 0.6	15.1 ± 0.3	8.5 ± 0.1	47 ± 6	2.0 ± 0.2	18.2
14.8 ± 0.7	2.32 ± 0.1	16.3 ± 0.2	6.4 ± 0.1	145 ± 7	2.9 ± 0.2	21.8

## Data Availability

No new data were created or analyzed in this study. Data sharing is not applicable to this article.

## Supplementary Materials

The following are available online at https://www.mdpi.com/article/10.3390/nano11061427/s1, Derivation of the Svedberg equation for NC@SL and Figure S1: 3D plot of s_AuNC@CTAB@W_ and (f/f_c_)_AuNC@CTAB@W_ (lower) distributions obtained at different wavelengths.
