# Supplementary material for: Simple Determination of Gold Nanocrystal Dimensions by Analytical Ultracentrifugation via Surface Ligand-Solvent Density Matching
Source: Nanomaterials (Basel). 2021 May 28;11(6):1427. doi: 10.3390/nano11061427 (PMC8228700; doi:10.3390/nano11061427)
Supplement: Supplementary file 1 [file nanomaterials-11-01427-s001.zip › nanomaterials-1207823-supplementary.pdf]

nanomaterials

MDPI

Supplementary Materials

Simple Determination of Gold Nanocrystal Dimensions by Analytical Ultracentrifugation via Surface Ligand-Solvent Density Matching

Guillermo González-Rubio *, Holger Hilbert, Rose Rosenberg, Bing Ni, Lisa Fuhrer and Helmut Cölfen *

Physical Chemistry Department of Chemistry, University of Konstanz, Universitätsstraße 10, Box 714, 78457 Konstanz, Germany; holger.hilbert@uni-konstanz.de (H.H.); rose.rosenberg@uni-konstanz.de (R.R.); bing.ni@uni-konstanz.de (B.N.); lisa.fuhrer@uni-konstanz.de (L.F.)

* Correspondence: guillermo.gonzalez-rubio@uni-konstanz.de (G.G.-R.); helmut.coelfen@uni-konstanz.de (H.C.)

1. Derivation of the Svedberg Equation for NC@SL@SS

The interplay of three forces governs the sedimentation behaviour of a NC@SL@SS: i) the applied centrifugal force ($F_{s,NC@SL@SS,}$), ii) the buoyant force ($F_{b,NC@SL@SS}$), and iii) the frictional force ($F_{f,NC@SL@SS}$) (eqs. 7, 8 and 9 in the main text). Note that all eqs. follow the indexation given in the main text.

$$F_{f,NC@SL@SS} = -f_{NC@SL@SS} \cdot u \tag{7}$$

$$F_{s,NC@SL@SS} = \omega^2 \cdot r \cdot (m_{NC} + m_{SL} + m_{SS}) \tag{8}$$

$$F_{b,NC@SL@SS} = -\omega^2 \cdot r \cdot \rho \cdot \left(m_{NC} \cdot \frac{1}{\rho_{NC}} + m_L \cdot \frac{1}{\rho_{SL}} + m_S \cdot \frac{1}{\rho_{SS}} \right) \tag{9}$$

During the sedimentation experiment, a balance between the three forces is reached (eq. 10 in the main text):

$$F_{s,NC@SL@SS} + F_{b,NC@SL@SS} = F_{f,NC@SL@SS} \tag{10}$$

and by insertion of (4), (7), (8), and (9) in (10):

$$\omega^2 \cdot r \cdot (m_{NC} + m_{SL} + m_{SS}) - \omega^2 \cdot r \cdot \rho \cdot \left(m_{NC} \cdot \frac{1}{\rho_{NC}} + m_{SL} \cdot \frac{1}{\rho_{SL}} + m_{SS} \cdot \frac{1}{\rho_{SS}} \right) = \frac{kT}{D_{NC@SL@SS}} \cdot u \tag{S1}$$

$$\omega^2 \cdot r \cdot \left[(m_{NC} + m_{SL} + m_{SS}) - \rho\left(m_{NC} \cdot \frac{1}{\rho_{NC}} + m_{SL} \cdot \frac{1}{\rho_{SL}} + m_{SS} \cdot \frac{1}{\rho_{SS}}\right)\right] = \frac{kT}{D_{NC@SL@SS}} \cdot u \quad \text{(S2)}$$

$$\frac{D_{NC@SL@SS}\left[(m_{NC} + m_{SL} + m_{SS}) - \rho\left(m_{NC} \cdot \frac{1}{\rho_{NC}} + m_{SL} \cdot \frac{1}{\rho_{L}} + m_{SS} \cdot \frac{1}{\rho_{S}}\right)\right]}{kT} =\cdot \frac{u}{\omega^2 \cdot r} \cdot \quad \text{(S3)}$$

Where $u/\omega^2 r$ is the sedimentation coefficient of the NC@SL@SS, $s_{NC@SL@SS}$

$$s_{NC@SL@SS} = \frac{D_{NC@SL@SS}\left[(m_{NC} + m_{SL} + m_{S}) - \rho\left(m_{NC} \cdot \frac{1}{\rho_{NC}} + m_{L} \cdot \frac{1}{\rho_{L}} + m_{S} \cdot \frac{1}{\rho_{S}}\right)\right]}{kT} \quad \text{(S4)}$$

$$s_{NC@SL@SS} = \frac{D_{NC@SL@SS}\left[m_{NC} \cdot \left(1 - \frac{\rho}{\rho_{NC}}\right) + m_{SL} \cdot \left(1 - \frac{\rho}{\rho_{SL}}\right) + m_{SS} \cdot \left(1 - \frac{\rho}{\rho_{SS}}\right)\right]}{kT} \quad \text{(S5)}$$

Where $k=R/N_A$ (N_A = Avogadros constant), and thus, we arrive at the final form of the Svedberg equation for NC@SL@SS (11)

$$s_{NC@SL@SS} = \frac{D_{NC@SL@SS} \cdot N_A\left[m_{NC} \cdot \left(1 - \frac{\rho}{\rho_{NC}}\right) + m_{SL} \cdot \left(1 - \frac{\rho}{\rho_{SL}}\right) + m_{SS} \cdot \left(1 - \frac{\rho}{\rho_{SS}}\right)\right]}{R \cdot T} \quad \text{(11)}$$

And since $M = m\ N_A$

$$s_{NC@SL@SS} = \frac{D_{NC@SL@SS} \cdot \left[M_{NC} \cdot \left(1 - \frac{\rho}{\rho_{NC}}\right) + M_{SL} \cdot \left(1 - \frac{\rho}{\rho_{SL}}\right) + M_{SS} \cdot \left(1 - \frac{\rho}{\rho_{SS}}\right)\right]}{R \cdot T} \quad \text{(S6)}$$

Or

$$[M_{NC} \cdot (1 - \bar{v}_{NC}\,\rho) + M_{L} \cdot (1 - \bar{v}_{SL}\,\rho) + M_{S} \cdot (1 - \bar{v}_{SS}\,\rho)] = \frac{s_{NC@SL@SS} \cdot R\,T}{D_{NC@SL@SS}} \quad \text{(S7)}$$

2. 3D Plot of Sedimentation Coefficient and $(f/f_c)_{AuNC@CTAB@W}$ (lower) Distributions Obtained at Different Wavelengths.

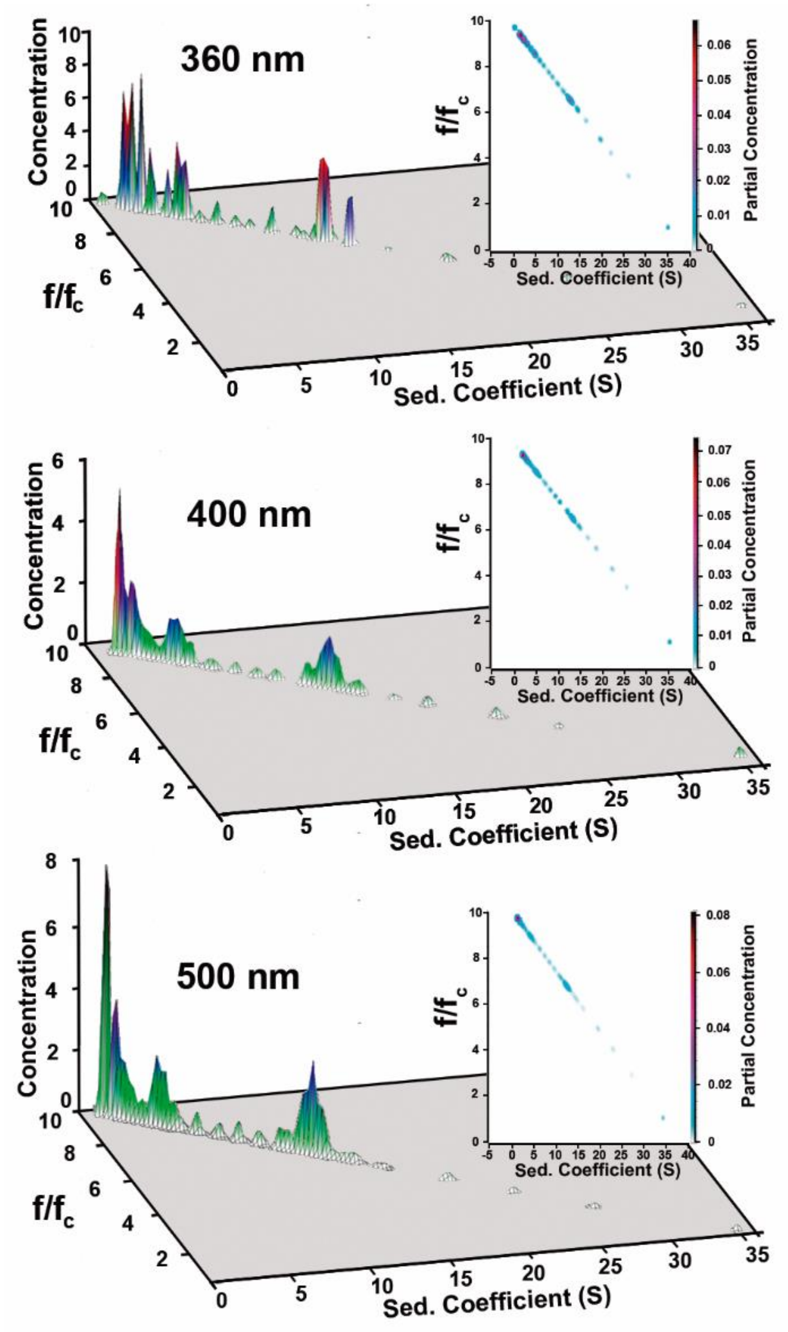

Figure S1. 3D and 2D (inset) plots of sedimentation coefficient and $(f/f_c)_{AuNC@CTAB@W}$ (lower) distributions obtained from sedimentation velocity AUC measurements at 360, 400 and 500 nm (using the density of Au to perform the analysis).

© 2021 by the authors. Licensee MDPI, Basel, Switzerland. This article is an open access article distributed under the terms and conditions of the Creative Commons Attribution (CC BY) license (http://creativecommons.org/licenses/by/4.0/).
